# Psychological Distress Among Older Adults During the First Wave of SARS-CoV-2 Pandemic: Survey of Health, Ageing, and Retirement in Europe

**DOI:** 10.3389/ijph.2023.1604372

**Published:** 2023-02-15

**Authors:** Orjola Shahaj, Gabriela Ksinan Jiskrova, Martin Bobák, Hynek Pikhart, Albert J. Ksinan

**Affiliations:** ^1^ RECETOX, Masaryk University, Brno, Czechia; ^2^ Institute of Epidemiology and Health Care, University College London, London, United Kingdom

**Keywords:** mental health, older adults, COVID-19, psychological distress, SHARE

## Abstract

**Objective:** To investigate the individual and country-level characteristics associated with the presence and worsening of psychological distress during the first wave of the pandemic among the elderly in Europe.

**Methods:** In June-August 2020, 52,310 non-institutionalized people aged 50+ in 27 SHARE participating countries reported whether feeling depressed, anxious, lonely, and having sleep problems. For this analysis, we combined these symptoms into a count variable reflecting psychological distress. Binary measures of the worsening of each symptom were used as secondary outcomes. Multilevel zero-inflated negative binomial and binary logistic regressions were used to assess the associations.

**Results:** Female sex, low education, multimorbidity, fewer social contacts, and higher stringency of policy measures were associated with increased distress. The worsening of all 4 distress symptoms was associated with younger age, poor health, loss of work due to the pandemic, low social contact, and high national mortality rates from COVID-19.

**Conclusion:** The pandemic exacerbated distress symptoms for socially disadvantaged older adults and those who were already struggling with mental health. The death toll of COVID-19 in a country played a role in symptom worsening.

## Introduction

The Sars-Cov-2 pandemic (COVID-19) has adversely affected societies worldwide. In addition to direct effects on health, important implications have arisen from the enforced restrictions (i.e., workplace closures, school closures and travel bans) that governments have implemented to contain the spread of the virus (1). The effects of these restrictions on mental health have been of particular concern (2–4). Some authors report increases in the prevalence of depressive symptoms, anxiety, posttraumatic stress symptoms, negative emotions, and psychological distress compared to pre-pandemic levels in the general population and some subgroups such as frontline health workers, students and the elderly (5–8).

The mental health of older adults (60+) has received substantial attention since they were classified as at increased risk for morbidity and mortality from COVID-19 (9–15). From the early stages of the pandemic, older persons were instructed to isolate and avoid face-to-face social contact. This affected many aspects of their daily lives, including loss of work for economically active older adults, reduced use of healthcare, increased loneliness, and lower quality of life (13, 16, 17). The extended periods of isolation worsened their mobility and movement, leading to deconditioning, muscle weakeness and joint pain, which makes performing daily life activities even more challenging. The lack of socialising and mental stimulation has disproportionately affected older adults, raising concerns about cognitive decline. Before the pandemic, it was estimated that approximately 15% of people over 60 years of age suffered from some form of mental illness (18), and the COVID-19 pandemic could plausibly worsen their mental health.

Several cross-sectional studies have identified factors associated with exacerbated psychological distress during the pandemic, such as female sex, pre-existing mental health conditions, concerns about getting infected, and poor self-rated health (7, 19–21). However, these findings come from small studies, often using non-representative samples and focusing on specific populations, such as healthcare workers, adolescents, and students, while relatively few large population-based studies of older adults have been reported (22–24). In addition to individual characteristics, it is possible that contextual factors such as a country’s economic development, inequality and pandemic response also affected the mental health of populations. There was substantial variation between countries regarding the onset of the pandemic, its severity, and the restrictive measures that followed (25).

We conducted a secondary analysis of data collected by the Survey for Health, Ageing and Retirement in Europe (SHARE) (26) to investigate individual and country-level factors associated with the presence and worsening of depressive symptoms among older adults living in 27 countries across Europe.

## Methods

### Study Design

SHARE is a cross-national panel database of data on health, socioeconomic status, and social networks of non-institutionalized individuals aged 50 years or older selected from the population registers in 27 European countries and Israel. [ref] Since its setup, new countries and refreshment samples have been added to each wave, which means that not all participants have been followed longitudinally. Further details of the SHARE study are described elsewhere (27). We used data from wave 8 of SHARE, which focused on the impact of the COVID-19 pandemic in older adults (28). Computer-assisted telephone interviews (CATI) were conducted between June and August 2020, and 52,310 panel members were interviewed (27).

The Ethics Council of the Max-Planck Society has granted the ethical approval of waves 5–8, whereas previous ones are approved by the Ethics Committee at the University of Mannheim. Ethics committees or institutional review boards have approved the implementation of SHARE in participating countries, and all participants gave their informed consent.

### Outcomes

Participants were asked if in the 4 weeks preceding the interview, they felt sad/depressed, nervous, lonely, and if they had trouble sleeping. Those who answered yes to any of these four questions were further probed whether the particular problem worsened, improved or remained the same compared to before the pandemic.

From these questions, we derived two sets of outcomes. First, a variable called “psychological distress” was generated as the primary outcome. It represents the count of the four symptoms described above, ranging from 0 to 4. Although three of these questions are items of the “Euro-Depression Screening Scale” (EURO-D), we called this variable “*psychological distress*” rather than “depression” because only a small portion of the questionnaire was administered.

Second, for those who reported the presence of any of the four symptoms above, we used information on the worsening of these symptoms (i.e., responses *“More depressed,” “More nervous,” “More trouble sleeping,” and “Lonelier”*) as four separate variables (secondary outcomes). We could not combine them into a single variable (as we did for the primary outcome) because each was derived from a different subsample. These variables are coded as binary, where 1 indicates worsening of the problem during the pandemic and 0 means that it remained the same or improved.

Please note that, given the survey design, the number of responses differs between the analyses of the primary and secondary outcomes (see below).

### Independent Variables

#### Individual-Level

##### Sociodemographic Variables

*Age* at the day of the interview (in years) was classified into 4 age groups (<60, 61–70, 71–80, and 80+). *Sex* was coded as binary, 0 = male and 1 = female. *Having a partner* or not, regardless of whether the partner lives in the household, was coded as binary (yes/no). *Living alone* in the household during the COVID-19 pandemic was also coded as binary (yes/no). Information on *education level* was retrieved from previous waves (it was not asked in the COVID-19 Survey), and it was coded as follows: 0 = tertiary, 1 = secondary, 2 = primary. Similarly, the number of chronic diseases with which a participant lives was recovered from wave 7, and a *multimorbidity* variable was derived, coded as 2+ conditions vs. ≤2.

##### Pandemic-related variables

*The self-perceived change in overall health during* the pandemic was coded as improved/same vs. worsened. *Job loss due to COVID-19*, *knowing someone hospitalized with COVID-19* (children, parents, relatives, colleagues, friends, neighbours, and others), and *knowing someone who died due to COVID-19* are also binary variables, coded yes vs. no*. The frequency of social contact*, including in-person and electronic contact with children, parents, relatives, colleagues, friends, neighbours, or others, was coded as every day, several times a week, once a week, and less often than once a week.

#### Country-Level Variables

We also used several country-level variables. *Covid deaths per million* w retrieved from the online database “Our World In Data” and indicates the total number of COVID-19 attributed deaths per 1 million inhabitants on each country’s last day of the SHARE fieldwork. The *Stringency Index*, also taken from “Our World In Data,” is a composite measure based on nine response indicators, including school closures, workplace closures, and travel bans, rescaled to a value of 0–100 (100 = strictest). If policies vary at the subnational level, the index refers to the strictest subregion (29). *GINI coefficient of income inequality* was retrieved from the Eurostat website using 2018 data (30). Finally, *GDP per capita adjusted for purchasing power parity (USD PPP)* was retrieved from the World Bank website and refers to the year 2019 (31).

### Analytical Samples

A total of 51,582 participants had complete data on all four mental health questions in 2020. There were very few missing values in the primary outcome, *psychological distress* (1%), but the analytical sample of the primary outcome was reduced to 44,841 due to missing values in the independent variables (most missing values were observed for living with chronic conditions—12%). For the secondary outcomes (*worsening of depressive symptoms during the pandemic*), the numbers were much smaller: 13,447 for feeling more depressed; 15,687 for feeling more nervous; 14,463 for worsening sleep; and 14,861 for feeling lonelier. This is because only participants who reported the presence of a symptom were asked if that symptom worsened during the pandemic. After excluding observations with missing values in the independent variables, the analytical samples for the secondary outcomes were reduced to 11,800; 13,755; 12,897, and 13,001, respectively.

### Statistical Analysis

The statistical analysis had two parts with different sample sizes, depending on the dependent variable. The first part investigated *psychological distress* as the outcome (*n* = 44,841), while the second part focused on the worsening of the four symptoms during the pandemic with smaller sample sizes ranging between 11,800 and 13,755 (see above). In both parts of the analyses, mixed-effects models were used to account for the hierarchical design of the data, where individuals are nested into households and countries.

For the primary outcome (*psychological distress*), the analysis was performed in “MPlus” using *multilevel zero-inflated negative binomial regression (ZINB);* only nesting within countries was considered. The choice of *zero-inflated negative binomial* was guided by comparing it with negative binomial and zero-inflated Poisson, using log-likelihood and the Akaike Information Criterion. ZINB produces two estimates: an odds ratio (OR) for having at least one symptom and a rate ratio (RR) for the overall count of symptoms in the presence of some symptoms (in both cases comparing each exposure category with the reference group).

For the secondary outcomes (*worsening of symptoms*), the analysis was carried out using multilevel logistic regression in Stata/IC version 16.1.

Missingness was dealt with listwise deletion because most missing values arose from the predictor variables. The baseline characteristics of the observations that were excluded from the model were compared with those that were included.

## Results

Table 1 describes the total analytical sample. The largest age group was 61–70 years (37%), followed by those aged between 71 and 80 (32%), and 58.5% of the participants were women. In addition, 36% of the respondents had in-person or electronic social contacts less than once a week, and 25% lived alone. Only 3.5% reported that they or someone they knew was hospitalized with COVID-19, while less than 3% reported that they knew someone who had died of COVID-19. Around 15% of the participants lived with more than 2 chronic conditions, and 9% said their health worsened during the COVID-19 pandemic.

**TABLE 1:** Descriptive characteristics of the analytical study sample (Survey for Health, Ageing and Retirement in Europe, First COVID-19 wave, June-August 2020, 27 European Countries).

Variable	n	%/Mean (SD)	Min	Max
Age				
<60	6,690	14.9%		
61–70	16,731	37.3%		
71–80	14,280	31.8%		
80+	7,140	15.9%		
Female	26,245	58.5%		
Education				
Tertiary	15,275	34.1%		
Secondary	19,234	42.9%		
Primary	10,332	23.0%		
Has a partner %	30,736	68.5%		
Alone in the household	11,091	24.7%		
Multimorbidity	6,895	15.4%		
Worse health after the pandemic	4,099	9.1%		
Lost job due to Covid	1,726	3.8%		
Someone close hospitalized with Covid	1,569	3.5%		
Someone close died of Covid	1,161	2.6%		
Frequency of social contact				
Daily	10,910	24.3%		
Several times a week	9,590	21.4%		
Once a week	8,017	17.9%		
Less often	16,324	36.4%		
Count of distress symptoms				
None	18,957	42.3%		
1 symptom	11,336	25.3%		
2 symptoms	6,745	15.0%		
3 symptoms	4,885	10.9%		
4 symptoms	2,918	6.5%		
More depressed (*N = 11,800*)	7,414	62.8%		
More nervous (*N = 13,755*)	9,725	70.7%		
More trouble sleeping (*N = 12,897*)	3,735	29.0%		
Lonelier (*N = 13,001*)	5,208	40.1%		
Total COVID-19 deaths per million	27	30.1 (4.25)	22.8	40.8
Stringency Index	27	57 (6.82)	41.6	69.4
GDP/capita (USD PPP)	27	36,360 (14,799)	18,563	94,278
GINI	27	185 (230)	5.1	847

Of the total sample, 58% of the respondents reported having at least one symptom. The prevalence of at least one symptom ranged from 43% in Switzerland to 73% in Portugal. Only 6% of the participants reported having all four symptoms; this proportion ranged from 1% in countries like the Netherlands or Denmark to 12% in Portugal. Supplementary Tables S1, S2 show the prevalence of primary and secondary outcomes by country.

Regarding the worsening of symptoms among participants who reported a given symptom, 63% of those who reported feeling depressed said they felt more depressed than before the pandemic, 71% felt more nervous, 29% had more trouble sleeping, and 40% felt lonelier. The worsening of symptoms varied substantially between countries.

At the country level, the Stringency Index ranged from 41.6 to 69.4, and the COVID-19 deaths per million ranged from 22.8 to 40.8. Likewise, GDP/capita varied from 18,563 to 94,278 USD PPP (adjusted for purchasing power parity). Important variations were also observed for the GINI index, which varied between 5.1 and 847.

Table 2 shows the association of *psychological distress* with predictor variables in the total sample. There were no statistically significant differences in the rate of distress symptoms between age groups, but those over 80 were twice as likely to report having at least one symptom compared to people <60 years of age (OR = 2.00).

**TABLE 2:** Multivariate adjusted odds ratios (OR) and rate ratios (RR) from multilevel zero-inflated negative binomial regression for the count of psychological distress symptoms: depressed, nervous, trouble sleeping, and lonely (N = 44,841) (Survey for Health, Ageing and Retirement in Europe, First COVID-19 wave, June-August 2020, 27 European Countries).

Fixed effects: Psychological distress	Odds ratio^a^ (95% CI)	P-value	Rate ratio^b^ (95%CI)	P-value
Female	1.90 (1.43–2.52)	<0.001	1.24 (1.18–1.32)	<0.001
Age group				
<60	*-ref-*	*-ref-*	*-ref-*	*-ref-*
60–69	1.15 (0.94–1.41)	0.174	0.97 (0.93–1.01)	0.118
70–71	1.26 (1.01–1.58)	0.044	0.98 (0.94–1.03)	0.448
80+	2.00 (1.12–3.59)	0.020	1.00 (0.94–1.07)	0.889
P-value for trend		0.001		0.306
Education level				
High	*-ref-*	*-ref-*	*-ref-*	*-ref-*
Middle	1.07 (0.93–1.23)	0.325	1.05 (1.01–1.10)	0.024
Low	1.23 (1.08–1.41)	0.002	1.13 (1.06–1.21)	<0.001
P-value for trend		0.002		<0.001
Having a partner	0.49 (0.27–0.87)	0.015	0.93 (0.87–0.99)	0.016
Living alone	1.65 (0.81–3.35)	0.167	1.08 (1.04–1.13)	<0.001
Multimorbidity	1.70 (1.17–2.48)	0.006	1.18 (1.13–1.23)	<0.001
Worsening health during pandemic^c^	8.28 (0.54–127.51)	0.13	1.56 (1.42–1.72)	<0.001
Lost job due to Covid	1.23 (0.96–1.58)	0.098	1.03 (0.97–1.09)	0.398
Someone hospitalised from Covid	1.33 (1.06–1.67)	0.014	1.03 (0.94–1.13)	0.54
Someone died from Covid	1.21 (0.84–1.74)	0.303	1.06 (0.99–1.14)	0.088
Frequency of social contact^d^				
Every day	*-ref-*	*-ref-*	*-ref-*	*-ref-*
Several times a week	1.11 (1.00–1.23)	0.042	1.04 (1.00–1.08)	0.058
Once a week	1.28 (1.06–1.53)	0.009	1.06 (1.01–1.10)	0.007
Less often	1.34 (1.11–1.62)	0.002	1.09 (1.04–1.13)	<0.001
P-value for trend		0.005		<0.001
Country-level				
Covid deaths per million (/100)	0.99 (0.96–1.02)	0.383	1.00 (0.99–1.02)	0.644
Stringency index (/10)	1.04 (0.81–1.33)	0.755	1.08 (1.01–1.15)	0.031
GDP *per capita* ($ PPP)^e^ (/10,000)	0.99 (0.91–1.07)	0.74	0.97 (0.93–1.02)	0.216
GINI^e^(/10)	1.08 (0.85–1.37)	0.528	1.06 (0.97–1.17)	0.201

^a^ Odds of having at least one symptom.

^b^ Incidence rate ratio of having one more symptom.

^c^ Self-perceived health.

^d^ Including electronic contact.

^e^ Grand-mean cantered.

Female sex, low level of education (primary), multimorbidity, and low frequency of social contact were all associated with increased odds of reporting at least 1 symptom (OR = 1.9; OR = 1.23; OR = 1.7; OR = 1.34), as well as with a higher count of symptoms (RR = 1.24; RR = 1.13; RR = 1.18; RR = 1.09). Having a partner was found to have a protective effect-lower odds of reporting any symptoms (OR = 0.49) and lower rates of symptom count (RR = 0.93). Living alone (RR = 1.06) and worsening of health during the pandemic (RR = 1.56) were associated with a higher count of distress symptoms.

Losing the job due to the pandemic and knowing someone who died from it were not associated with the presence of distress symptoms but knowing someone hospitalized with COVID-19 increased the odds of reporting at least one symptom (OR = 1.33).

From the country-level variables, only the Stringency Index was associated with a significant but small effect on the rate of distress (RR = 1.08); we did not find associations with the other national characteristics.

The results about symptoms worsening (based only on those who reported the presence of symptoms) are shown in Table 3.

**TABLE 3:** Multivariate adjusted odds ratios (OR) for multilevel binary logistic regressions about worsening of four distress symptoms (depressed, nervous, trouble sleeping, and lonely) during the first wave of the Sars-Cov-2 pandemic (Survey for Health, Ageing and Retirement in Europe, First COVID-19 wave, June-August 2020, 27 European Countries).

Fixed effects	More depressed than before the pandemic N = 11,800		More nervous than before the pandemic N = 13,755		More trouble sleeping than before the pandemic N = 12,897		Lonelier than before the pandemic N = 13,001	
	OR (95% CI)	P>|z|	OR (95% CI)	P>|z|	OR (95% CI)	P>|z|	OR (95% CI)	P>|z|
Female sex	1.48 (1.30–1.69)	<0.001	1.31 (1.17–1.48)	<0.001	1.09 (0.97–1.23)	0.139	1.59 (1.41–1.81)	<0.001
Age group <60	*-ref-*	*-ref-*	*-ref-*	*-ref-*	*-ref-*	*-ref-*	*-ref-*	*-ref-*
61–70	0.87 (0.71–1.06)	0.169	0.86 (0.71–1.03)	0.109	0.70 (0.58–0.84)	<0.001	1.23 (1.00–1.50)	0.046
71–80	0.68 (0.55–0.85)	0.001	0.72 (0.59–0.87)	0.001	0.55 (0.45–0.67)	<0.001	1.13 (0.92–1.39)	0.254
80+	0.55 (0.43–0.69)	<0.001	0.46 (0.36–0.58)	<0.001	0.35 (0.28–0.45)	<0.001	1.08 (0.86–1.36)	0.51
P-value for trend		*<0.001		*<0.001		*<0.001		*0.829
Education level								
Tertiary	*-ref-*	*-ref-*	*-ref-*	*-ref-*	*-ref-*	*-ref-*	*-ref-*	*-ref-*
Secondary	1.11 (0.96–1.29)	0.155	1.34 (1.16–1.55)	<0.001	1.18 (1.02–1.36)	0.024	1.09 (0.95–1.26)	0.214
Primary	1.19 (0.99–1.43)	0.065	1.53 (1.28–1.82)	<0.001	1.22 (1.03–1.45)	0.020	1.16 (0.98–1.38)	0.089
P-value for trend		*0.053		*<0.001		*0.013		*0.067
Having a partner	1.65 (1.31–2.07)	<0.001	1.37 (1.09–1.71)	0.007	0.95 (0.76–1.18)	0.641	1.81 (1.45–2.25)	<0.001
Living alone in the household	1.00 (0.80–1.27)	0.971	1.26 (1.00–1.61)	0.054	0.95 (0.75–1.19)	0.632	1.34 (1.08–1.66)	0.008
Multimorbidity	0.92 (0.80–1.07)	0.295	0.84 (0.73–0.97)	0.018	1.05 (0.91–1.21)	0.495	1.05 (0.91–1.21)	0.515
Worsening health during pandemic^a^	3.73 (3.09–4.50)	<0.001	2.43 (2.06–2.88)	<0.001	4.57 (3.80–5.49)	<0.001	3.54 (2.95–4.25)	<0.001
Lost job due to Covid	2.79 (1.92–4.07)	<0.001	2.06 (1.49–2.85)	<0.001	1.79 (1.33–2.40)	<0.001	1.64 (1.18–2.27)	0.003
Someone hospitalized with Covid	1.39 (0.95–2.02)	0.088	1.74 (1.21–2.51)	0.003	1.30 (0.94–1.79)	0.113	1.41 (1.00–2.01)	0.053
Someone died from Covid	1.54 (1.01–2.35)	0.044	1.13 (0.76–1.68)	0.553	1.55 (1.07–2.25)	0.021	1.46 (0.98–2.18)	0.066
Frequency of social contact^b^								
Every day	*-ref-*	*-ref-*	*-ref-*	*-ref-*	*-ref-*	*-ref-*	*-ref-*	*-ref-*
Several times a week	1.35 (1.12–1.62)	0.002	1.17 (0.98–1.39)	0.087	1.12 (0.94–1.33)	0.198	1.42 (1.18–1.70)	<0.001
About once a week	1.21 (1.00–1.47)	0.047	1.09 (0.91–1.31)	0.356	1.03 (0.86–1.24)	0.726	1.80 (1.49–2.18)	<0.001
Less often	1.42 (1.21–1.68)	<0.001	1.34 (1.14–1.57)	<0.001	1.33 (1.14–1.55)	<0.001	1.94 (1.64–2.29)	<0.001
P-value for trend		*<0.001		*<0.001		*<0.001		*<0.001
Country-level variables								
Covid deaths per million^c^ (/100)	1.19 (1.09–1.30)	<0.001	1.15 (1.03–1.28)	0.011	1.19 (1.08–1.32)	0.001	1.14 (1.01–1.28)	0.028
Stringency index^c^ (/10)	1.29 (0.93–1.78)	0.129	1.11 (0.76–1.62)	0.600	1.39 (0.97–1.99)	0.069	1.30 (0.87–1.96)	0.206
GDP *per capita* ($ PPP)^c^ (/10,000)	1.14 (1.01–1.29)	0.037	1.19 (1.02–1.37)	0.022	0.98 (0.85–1.12)	0.768	1.25 (1.07–1.47)	0.005
GINI^c^ (/10)	1.56 (0.95–2.55)	0.081	1.31 (0.73–2.34)	0.363	1.13 (0.66–1.93)	0.667	0.93 (0.50–1.73)	0.829
Intercept (log odds)	0.02 (0.00–0.27)	0.003	0.17 (0.01–2.92)	0.220	0.02 (0.00–0.32)	0.005	0.01 (0.00–0.17)	0.002

^a^ Self-perceived health.

^b^ Including electronic contact.

^c^ Grand-mean centred.

Worsening health during the pandemic, losing a job, and having less than one social contact per week were risk factors for worsening all 4 symptoms (depression, nervousness, sleep problems, and loneliness). Worsening health during the pandemic displayed the highest effect sizes (OR = 3.73; OR = 2.43; OR = 4.57; OR = 3.54). Female sex was associated with a higher likelihood of reporting worsening depression, anxiety, and loneliness, but not sleep problems.

Compared to people aged <60, older adults aged 61–70 and 81–90 were less likely to report an exacerbation of feeling depressed, anxious, or having trouble sleeping. They were slightly more likely to report feeling lonelier, but it was not statistically significant (*p* = 0.25; *p* = 0.51).

Among country-level variables, higher deaths from COVID-19 per million were associated with higher odds of observing worsening of all four outcomes. Higher Stringency Index and higher GINI were also associated with higher odds of symptom worsening, but these associations were not statistically significant. Unexpectedly, higher GDP *per capita* yielded some statistically significant association with higher odds of symptoms worsening.

Supplementary Table S3 summarizes the comparison of baseline characteristics between observations that were excluded from the model (due to missing values in the predictors) with those that were included in the model. The two groups were largely similar, and the differences are very small, but the comparison tests yielded significant results. This may be due to an overpowered sample.

## Discussion

This study of psychological distress during the first wave (Spring 2020) of the COVID-19 pandemic in 27 European countries found that individual-level factors associated with the presence and worsening of distress symptoms were generally similar to those reported in the literature before the pandemic (32), including female gender, lower education, few social contacts, and poor physical health. In addition to these “usual suspects,” job loss due to Covid, living in a country with high Covid-related mortality, and stricter restrictions were also associated with distress. Interestingly, older adults did not show higher rates of distress symptoms and were less likely to report worsening of symptoms compared to their younger counterparts. Country-level characteristics had little to no effect on the presence of symptoms but played a role in their worsening.

Several of our findings should be seen in the context of previous reports. First, the high prevalence of low frequency of social contact (37% of SHARE participants reported having less than one social contact per week) is of concern. In this analysis, we noticed a stepwise association, where decreasing frequency of social contact is linked to increased rates of distress and a higher likelihood of symptom worsening. This finding has important practical implications, and adequate public health interventions should be implemented to reduce or mitigate the harmful effects of loneliness.

Second, persons aged 70–79 and 80+ had similar rates of distress symptoms as those aged <60, but they were less likely to have reported worsening symptoms. This protective effect of older age suggests that they may be more resilient in the face of adversity. A systematic review of 20 studies that measured the emotional resilience of older adults during the pandemic concluded that older adults had fewer negative emotions during quarantine (33). Such findings contradict the assumption that older adults would be more vulnerable during the first wave (34).

A longitudinal analysis of pre-and post-pandemic depressive symptoms from the “UK National Household Panel Study” assessed participants’ mental health through a 12-item General Health Questionnaire (GHQ-12). It found that during the first wave of the pandemic, older adults (>70) had a lower mean score of depressive symptoms compared to people <45, and women scored higher than men (35). This corroborates our findings. However, in the same study, people with higher education levels were disproportionally affected by psychological distress. Our data point in a different direction and suggest that people with low education reported higher rates of distress symptoms and were more likely to have experienced worsening symptoms during the first wave of the pandemic. This contradictory finding could be partially explained by the fact that different tools were used to measure psychological distress.

Third, we found that living with several chronic diseases and worsening health during the pandemic were associated with the presence of more symptoms and worsening mental health. Similar results were found in the ELSA COVID Study (24). This could be due to many factors, including restricted access to healthcare services and increased worry over physical health. Therefore, policymakers must acknowledge that older adults with multimorbidity are more susceptible to mental health deterioration.

Fourth, our results suggest that people who already experienced some or all of the distress symptoms were most affected during the first wave of the pandemic (March to August 2020). Another article that used SHARE data reported a similar finding, where people who were depressed or anxious in a previous SHARE wave were positively associated with “…change for the worse in each mental health outcome after the COVID-19 pandemic” (22). However, a case-control study in the Netherlands with participants from 3 ongoing mental health cohorts found the opposite (36). The study concluded that the severity of symptoms among those who already suffered from depression, anxiety, or OCD was not notably higher during the pandemic. This finding points in a different direction from ours, although we cannot be entirely sure whether our participants had any established condition or not, as the questionnaire is not a clinical diagnosis.

Fifth, higher GDP/capita appeared to be related to the worsening of three symptoms. This is possibly a reflection of the dynamics of the first wave of the pandemic, where Western European countries with higher GDP/capita were hit harder than some Central and Eastern European countries (25). Contrary to our expectations, the Stringency Index did not yield statistically significant associations with worsening of the symptoms. This may reflect the flaws in how this composite measure is constructed, which may not accurately capture the state of the restrictions across Europe.

Finally, the evidence on the impact of some country-level variables on the worsening of symptoms during the pandemic may suggest that the strong restrictive measures (i.e., enforced isolation, workplace closures, and travel bans) affected more those who were already struggling with their mental health. However, individual-level characteristics predicted distress better than country-level variables. In a similar analysis of SHARE COVID-19 performed only for the symptom of feeling sad/depressed, Atzendorf and Gruber found similar results (23).

### Strengths and Limitations

This study benefited from large nationalprobability samples. Including populations of older adults from 27 different countries allowed us to assess the effects of contextual characteristics, providing some further clarity on a highly contentious topic such as the impact of lockdowns on mental health.

Our study also has several limitations. First, the results may not accurately capture the real impact of the first wave of COVID-19 on the elderly because the “SHARE COVID-19” interviews were conducted towards the end of 2020 summer. During this time, the pandemic was subsiding in most European countries, and restrictions were lifted, which may have led to a temporary amelioration in mental health. In a paper from the “UCL COVID-19 Social Study,” where depression and anxiety were measured weekly for more than 36,000 participants, the highest levels of distress were reported in the first 2 weeks after the lockdown was announced, and it decreased afterwards as people adapted to restrictions (37). Similar trends are seen in other studies (38, 39), pointing to an episodic nature of distress symptoms that may also have played a role in SHARE.

Second, symptoms were self-reported, making them susceptible to information bias. Furthermore, the questionnaire used only 3 items from the EURO-D scale and 1 loneliness question, which we combined into a measure of psychological distress that may not capture all aspects of depression as intended by the EURO-D scale.

Third, for the analysis of the “worsening” of depressive symptoms, we had 4 different analytical samples, making it difficult to compare the results for these outcomes directly. The statistical models included only random intercepts and did not allow slopes to vary. Consequently, we did not test cross-level interactions, and further analysis may be needed to understand the effects of country-level variables on mental health.

Finally, the response rates in the SHARE project are modest and differ between countries; therefore, the findings may not be fully representative of their respective national populations, which may also affect the estimated associations between distress and country-level characteristics.

### Conclusion

The first wave of the COVID-19 pandemic and the restrictions that accompanied it were expected to have a detrimental effect on the mental health of older adults. However, we noticed that overall, older adults were resilient to worsening distress symptoms during the first wave. However, several individual factors may have exacerbated the psychological distress in this population, and these factors (including impaired physical health, job loss, and low social contact) may need to be addressed by appropriate policies.
