# Supplementary material for: Psychological Distress Among Older Adults During the First Wave of SARS-CoV-2 Pandemic: Survey of Health, Ageing, and Retirement in Europe
Source: Int J Public Health. 2023 Feb 15;68:1604372. doi: 10.3389/ijph.2023.1604372 (PMC9974635; doi:10.3389/ijph.2023.1604372)
Supplement: Supplementary file 1 [file DataSheet1.docx]

**Supplementary Table 1** - Distribution of the count of distress symptoms by country. [Survey for Health, Ageing and Retirement in Europe, First COVID-19 wave, June-August 2020, 27 European Countries]

| **Country** | **Number of distress symptoms (n, %)** | | | | | |
| --- | --- | --- | --- | --- | --- | --- |
|  | **N=51,582** | | | | | |
|  | *None* | *At least one* | 1 | 2 | 3 | 4 |
| *Germany* | 1,222 (46%) | 1,421 (54%) | 692 (26%) | 371 (14%) | 244 (9%) | 114 (4%) |
| *Sweden* | 684 (51%) | 665 (49%) | 355 (26%) | 177 (13%) | 98 (7%) | 35 (2%) |
| *Netherlands* | 434 (56%) | 345 (44%) | 193 (24%) | 85 (10%) | 52 (6%) | 15 (1%) |
| *Spain* | 857 (43%) | 1,135 (57%) | 431 (21%) | 298 (14%) | 266 (13%) | 140 (7%) |
| *Italy* | 1,318 (36%) | 2,369 (64%) | 882 (23%) | 608 (16%) | 502 (13%) | 377 (10%) |
| *France* | 787 (39%) | 1,226 (61%) | 509 (25%) | 330 (16%) | 252 (12%) | 135 (6%) |
| *Denmark* | 1,102 (56%) | 874 (44%) | 503 (25%) | 253 (12%) | 95 (4%) | 23 (1%) |
| *Greece* | 1,483 (41%) | 2,128 (59%) | 929 (25%) | 489 (13%) | 443 (12%) | 267 (7%) |
| *Switzerland* | 1,065 (57%) | 810 (43%) | 438 (23%) | 204 (10%) | 115 (6%) | 53 (2%) |
| *Belgium* | 1,585 (42%) | 2,182 (58%) | 923 (24%) | 575 (15%) | 447 (11%) | 237 (6%) |
| *Israel* | 539 (39%) | 854 (61%) | 388 (27%) | 219 (15%) | 143 (10%) | 104 (7%) |
| *Czech Republic* | 1,208 (47%) | 1,372 (53%) | 672 (26%) | 353 (13%) | 213 (8%) | 134 (5%) |
| *Poland* | 1,025 (36%) | 1,853 (64%) | 738 (25%) | 529 (18%) | 359 (12%) | 227 (7%) |
| *Luxembourg* | 389 (43%) | 521 (57%) | 254 (27%) | 133 (14%) | 84 (9%) | 50 (5%) |
| *Hungary* | 426 (43%) | 566 (57%) | 232 (23%) | 136 (13%) | 125 (12%) | 73 (7%) |
| *Portugal* | 297 (27%) | 796 (73%) | 254 (23%) | 215 (19%) | 195 (17%) | 132 (12%) |
| *Slovenia* | 1,538 (50%) | 1,543 (50%) | 772 (25%) | 397 (12%) | 248 (8%) | 126 (4%) |
| *Estonia* | 1,664 (37%) | 2,817 (63%) | 1,238 (27%) | 769 (17%) | 527 (11%) | 283 (6%) |
| *Croatia* | 894 (45%) | 1,099 (55%) | 451 (22%) | 264 (13%) | 230 (11%) | 154 (7%) |
| *Lithuania* | 484 (38%) | 775 (62%) | 321 (25%) | 190 (15%) | 153 (12%) | 111 (8%) |
| *Bulgaria* | 325 (40%) | 480 (60%) | 214 (26%) | 128 (15%) | 88 (10%) | 50 (6%) |
| *Cyprus* | 373 (48%) | 404 (52%) | 175 (22%) | 100 (12%) | 72 (9%) | 57 (7%) |
| *Finland* | 719 (50%) | 723 (50%) | 356 (24%) | 218 (15%) | 113 (7%) | 36 (2%) |
| *Latvia* | 395 (41%) | 572 (59%) | 242 (25%) | 145 (14%) | 113 (11%) | 72 (7%) |
| *Malta* | 287 (35%) | 538 (65%) | 198 (24%) | 154 (18%) | 125 (15%) | 61 (7%) |
| *Romania* | 604 (41%) | 874 (59%) | 342 (23%) | 228 (15%) | 184 (12%) | 120 (8%) |
| *Slovakia* | 396 (42%) | 540 (58%) | 245 (26%) | 134 (14%) | 86 (9%) | 75 (8%) |
| **Total** | **22,100 (43%)** | **29,482 (57%)** | **12,947 (25%)** | **7,702 (14%)** | **5,572 (10%)** | **3,261 (6%)** |

**Supplementary Table 2** - Distribution of worsening outcomes by country. [Survey for Health, Ageing and Retirement in Europe, First COVID-19 wave, June-August 2020, 27 European Countries]

|  | **More depressed** | | **More nervous** | | **More sleep trouble** | | **Lonelier** | |
| --- | --- | --- | --- | --- | --- | --- | --- | --- |
| **Country** | % | n | % | n | % | N | % | N |
| *Germany* | 54% | 694 | 72% | 592 | 22% | 720 | 42% | 619 |
| *Sweden* | 68% | 262 | 75% | 314 | 33% | 212 | 60% | 357 |
| *Netherlands* | 75% | 151 | 87% | 145 | 36% | 121 | 60% | 164 |
| *Spain* | 74% | 672 | 79% | 790 | 45% | 513 | 36% | 463 |
| *Italy* | 77% | 1,267 | 77% | 1,381 | 52% | 972 | 49% | 1,496 |
| *France* | 68% | 599 | 74% | 660 | 37% | 610 | 40% | 622 |
| *Denmark* | 64% | 273 | 82% | 527 | 26% | 314 | 59% | 277 |
| *Greece* | 72% | 876 | 81% | 1,261 | 50% | 634 | 55% | 1,549 |
| *Switzerland* | 70% | 370 | 80% | 346 | 31% | 307 | 54% | 387 |
| *Belgium* | 78% | 1,005 | 81% | 1,216 | 41% | 1,055 | 59% | 1,095 |
| *Israel* | 70% | 343 | 71% | 439 | 31% | 472 | 48% | 435 |
| *Czech Republic* | 49% | 545 | 66% | 559 | 20% | 755 | 23% | 721 |
| *Poland* | 44% | 1,051 | 52% | 873 | 22% | 1,112 | 27% | 793 |
| *Luxembourg* | 71% | 250 | 80% | 291 | 26% | 231 | 54% | 209 |
| *Hungary* | 37% | 314 | 39% | 316 | 10% | 315 | 20% | 230 |
| *Portugal* | 68% | 475 | 67% | 560 | 27% | 438 | 40% | 335 |
| *Slovenia* | 51% | 571 | 66% | 744 | 22% | 813 | 31% | 708 |
| *Estonia* | 62% | 1,148 | 71% | 1,357 | 13% | 1,806 | 23% | 1,229 |
| *Croatia* | 53% | 493 | 58% | 583 | 27% | 557 | 31% | 663 |
| *Lithuania* | 63% | 353 | 75% | 459 | 26% | 446 | 26% | 348 |
| *Bulgaria* | 56% | 202 | 54% | 276 | 32% | 234 | 33% | 226 |
| *Cyprus* | 70% | 185 | 66% | 222 | 40% | 164 | 47% | 256 |
| *Finland* | 57% | 288 | 58% | 393 | 23% | 357 | 47% | 250 |
| *Latvia* | 49% | 197 | 70% | 291 | 26% | 333 | 21% | 353 |
| *Malta* | 80% | 266 | 77% | 419 | 38% | 192 | 48% | 250 |
| *Romania* | 59% | 415 | 61% | 471 | 24% | 512 | 28% | 433 |
| *Slovakia* | 49% | 212 | 54% | 202 | 38% | 268 | 28% | 393 |
| ***Total*** | **63%** | **13,477** | **71%** | **15,687** | **29%** | **14,463** | **40%** | **14,861** |

**Supplementary Table 3** - Comparison of baseline characteristics between participants that were included in the Zero-Inflated Negative Binomial Model and those that were excluded due to missing values in predictors. [Survey for Health, Ageing and Retirement in Europe, First COVID-19 wave, June-August 2020, 27 European Countries]

|  | **Included** | | **Excluded** | | **P-value** |
| --- | --- | --- | --- | --- | --- |
|  | *N* | *Mean (SD)/%* | *N* | *Mean (SD)/%* |  |
| *Age* | 44,841 | 70.5 (9.3) | 7,469 | 69.9 (9.9) | <0.001 |
| *Male* | 18,596 | 85 | 3,340 | 15 | <0.001 |
| *Female* | 26,245 | 87 | 3,933 | 13 |  |
| *Tertiary education* | 15,275 | 87 | 2,451 | 14 | <0.001 |
| *Secondary education* | 19,234 | 90 | 2,079 | 10 |  |
| *Primary education* | 10,332 | 88 | 1,347 | 12 |  |
